# Eco-Sustainability of the Textile Production: Waste Recovery and Current Recycling in the Composites World

**DOI:** 10.3390/polym13010134

**Published:** 2020-12-30

**Authors:** Antonella Patti, Gianluca Cicala, Domenico Acierno

**Affiliations:** 1 Department of Civil Engineering and Architecture (DICAr), University of Catania, Viale Andrea Doria 6, 95125 Catania, Italy; gianluca.cicala@unict.it; 2 CRdC Nuove Tecnologie per le Attività Produttive Scarl, Via Nuova Agnano 11, 80125 Naples, Italy

**Keywords:** textile productive process, environmental impact, recycling, composites

## Abstract

This work aimed to review the recent scientific research, focused on the application of recycled fibers, taken from textile waste, in the field of composite materials to fulfill the eco-sustainability requirements of textile manufacturing, and promote actions for a circular economy. The yarns and fabric production represent one of the most polluting processes of the industrial world. The harmful environmental impact of the textile process has been described by reporting the different treatments involving the raw material and the filament fabrication, and concerning the uses of insecticides, fertilizers, and many other chemicals for improving the quality of the final products. In addition, solid textile waste constituted a further additional issue for the environmental sustainability of fabric production. Various strategies have been discussed and in part already adopted by many companies to recover waste fibers and prevent them from ending up in landfills. The alternatives of fiber recycling for composite realization have been presented by reporting several recent studies involving the uses of recycled fibers from the textile waste embedded in different matrices: thermoplastic polymer, thermosetting resins, natural constituents, and concrete in light of specific applications.

## 1. Introduction

Since past ages, fabrics and fibers have always been an integral part of every human activity. We find the application of fabrics in clothing to protect and cover the human body from bad weather or simply to identify social status and respond to different fashions and trends. There are fabrics in each home for sheets and bed linen and towels, napkins, tea towels, table cloths, rugs, sofas, armchairs, chairs, curtains. There are fabrics in transport means, cars, trains, craft, airplanes for airbags, seat belts, carpets, seat upholstery, and tires. Fabrics are also used as structural reinforcement elements in construction fields [1].

Statistical studies by Garside M. (2019) [2] report that the production of chemical and textile fibers from 1975 to 2018 has increased approximately from 23.94 million metric tons to 105.6 million metric tons: this means an increment of more than four times in about 40 years.

As the demand for fabrics has grown, aspects linked to the environmental impact of fiber production, and the following disposal operations, have also become increasingly present. Numerous and recent studies show the danger of the textile industry for the freshwater and atmosphere micro-system, due to the usage of significant amounts of industrial harmful and toxic chemicals during the manufacturing process and the release of pollutants during the lifecycle of a textile product [3]. Other forms of contamination arise from high energy consumption, heavy transportation, and excessive packing material. Finally, given the huge waste generation, the problem of disposing of large solid volumes has arisen. Twenty percent of earth pollution has been attributed to the textile industry, which for these reasons has been considered among the most harmful and detrimental manufacturing processes for our ecosystem [4].

In the light of these complex ecological issues, over the years until 2020, European directives have promoted and encouraged the recovery of textiles in a board plan for new circular economic action for a cleaner and more competitive country [5].

One of the possible and potential applications of the recycled fibers is inherent to the world of composite materials: In this field, both synthetic and natural fibers have been just usefully incorporated into the matrix in order to enhance the characteristics of the final products, in terms of functional and structural features. In this direction, different attempts have been made by the scientific and academic communities through the combination of fibers of various nature, chemical composition, and size into the thermoplastics, thermosettings, or concrete in the light of distinct and specific applications. For example, natural fibers, mainly constituted by hemp, jute, flex, sisal, or cotton, have been added to the epoxy resin, or into the traditional polyolefins and biopolymers for aircraft and automotive structural applications, or for high-performance biodegradable products. Sustainable constructions were realized when natural reinforcement was introduced in the cementitious matrices. Synthetic fibers, the most common being glass and the carbon-based, have been used in thermosettings, in view of airplane interiors, military, or ballistic protection, as well as in polylactide resins for fused deposition models and additive manufacturing products. These types of fibers have been also utilized in concrete materials to provide the mechanical strength to the pavement or building constructions. Just to give an indicative idea of all the studies carried out on the use of fibers in the world of the composites, in Table 1, some examples have been reported.

Recently, research attention has been devoted to the possible reuse of industrial textile waste, or recovery of the used fabrics, for realizing recycled fibers, to be applied as fillers, in compounding with polymer or concrete matrices. In this way, the eco-sustainability of the textile manufacturing process and the realization of useful goods, adaptable to the different requirements, have been simultaneously satisfied.

In this framework, the aim of this work is to provide an overview of the current issues related to the environmental concerns of the textile productions, and of the alternative solutions for limiting the production of solid textile waste to be disposed. In detail, attention was devoted to collecting the most recent studies, written in the last decade, that dealt with the possible applications of the recycled fibers, coming from textile waste, into the world of composite materials.

## 2. Textile Fibrous Structures and Their Involvement in the Common and Technical Uses

The term textile is derived from the Latin “textilis” and the French “texere”, meaning “to weave”, and it originally referred only to woven fabrics; then, it was extended to fabrics produced by other methods, such as threads, cords, ropes, braids, lace, embroidery, nets, and fabrics made by weaving that are woven, knitted, braided, or non-woven [24].

Technically, the basic principle of weaving involves two series of filaments: the first one, called the warp, is pre-established and fixed, while the second, namely the weft, is in turn inserted within the first structure. The weaving process consists of three steps: (i) *shedding*, during which the warp sheet is divided into two patterns, one spaced, separated, and raised from the other so as to create an opening between the warp series; (ii) *filling insertion*, which provides for the insertion of a yarn inside the created opening; and (iii) *beat-up*, in which process the filling yarn is pushed inside the weave of the fabric [25].

**Figure 1:**
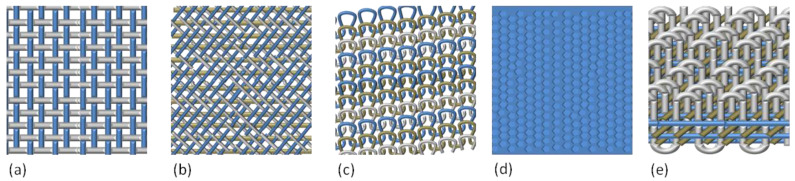
2D planar and 3D fibrous structures: (**a**) woven, (**b**) braid, (**c**) knit, (**d**) non woven, (**e**) tridimensional.

Through this method, 2D planar weaving structures, i.e., with in-plane oriented fibers, can be produced. Depending on the interlocking types, other conventional techniques exist, by which another kind of weaving, such as knitting, braiding, and non-woven fabrics, can be obtained. In detail, four main categories of textile constructions can be recognized (as seen in Figure 1): (i) woven, made by weaving two sets of filaments (warp and weft) by the orthogonal interlacement, continuously arranged at 0° and 90°; (ii) knitted fabrics, created by intermeshing loops and segments of fibers with different orientation; (iii) braided fabrics, in which the fibers are placed in the bias direction; and (iv) non-woven, in which the fibers are randomly oriented in the structure in a discontinuous way. In the nineteenth century, three dimensional (3D) structures from a set of fibers in the multiaxial orientation were developed, essentially made from fibers oriented in the three dimensions. These structures were born to meet the requirements imposed by aerospace’s industry of materials able to resist under multi-axial loads and severe thermal conditions; indeed, compared to 2D, the 3D textiles possess a higher mechanical resistance in x, y, and z directions [26].

The applications of each of the described types of fabric are the most varied, classified into products for clothing, household, and technical textiles [27]. In the case of woven fabrics, in the book of the Kim Ghandi [28], different technical applications for filtration in industry activities have been reported, as in the mechanical shaker cleaning method, together with air filtration in automobile engineers, or sound control and speak quality in the electronic devices (acoustic, loud-speakers, and microphones), liquid filtration techniques for chemicals in paper and pulp industry, and precipitation of metal oxides from solvent slurries. Typical structural components are made by 2D or 3D, woven, braided, knitted, or non-woven fabrics, such as knot elements (beams, shells, exhaust, seats, and chassis) or geotextiles (asphalt overlay, soil stabilization, drainage, sedimentation, and erosion control), to fulfill general requirements of easy manufacturability, low cost, sound and energy absorption, and corrosion resistance. In addition, all of them are utilized for ballistic applications as protective clothing or products, endowed with extremely high strength and modulus, ability in absorbing energy and sound, and in combating against the chemical and biological warfare agents. In aerospace applications, woven and braided fabrics are applied in soft space suits for astronauts, space shuttle components, and aircraft seat cushions, as well as in automobile interior for airbags or car seat parts [29].

## 3. Environmental Impact of the Textile Production

**Figure 2:**
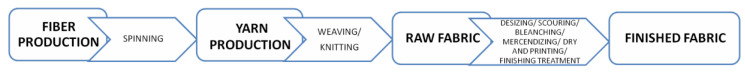
Typical progressive phases of the textile production.

The different steps starting from the fibers’ production that lead to the fabric realization can be described in the following operations: fiber production, yarn production, fabric production, pre-treatment, dyeing and printing, and finishing treatment. The common flow chart of the textile production starting from the fiber up to the finished fabric is reported in Figure 2.

As just predicted in the introduction, the environmental impact of these operations concern different aspects all connected to eachother that take into account the usage of harmful chemicals, the water and energy consumption, the air emission, the transportation, and the packaging.

The raw materials involved in the textile manufacturing are divided into two main categories: (i) natural fibers, deriving from vegetables and plants (cotton, flax, sisal, hemp, ramie, jute, banana, pineapple, coir, and oil palm), animals (wool, angora, cashmere, and silk), and minerals (asbestos); and (ii) man-made fibers, synthetic or regenerated, coming from petroleum-based resources, such as polyester, nylon, spandex, acrylic, and polypropylene. These primary materials, depending on their nature, require being treated with different chemical agents [30].

For instance, in the fiber production, pesticides, insecticides, and fertilizers are applied in the case of cellulose or natural fibers, to allow and facilitate the growth and development of plants, whereas for protein fibers, parasites are used for the animal, and after the fibers shredding, the utilization of chemicals for the cleaning operations [31]. In the case of the synthetic fibers, the preparation of the agents, polymerization, polymer recovery and extrusion, and spinning for arriving to the filaments required the use of monomers and catalyst that generate a series of byproducts [3].

Then, during the yarn production, particularly in the spinning, oil is required for reducing the friction among the parts, and again, in the fabric production, the sizing species together with the lubricants should be used for avoiding the breakage of the fibers during the process. Once obtained, and before the dyeing step, the fabric should be suitably prepared with a multiple pre-treatments through the following processes: (i) de-sizing, consisted in the removal of the starch (sizing chemical) and the improvement of the absorbent capability, since the starch hampers the diffusion of the dye molecule into the yarn/fabric; through enzymatic, or dilute mineral acid hydrolysis, or oxidation, the starch is transformed in water soluble constituents; (ii) scouring, during which wax, fats, pectin, and lubrication oil are removed by using aqueous sodium hydroxide together with the surfactant; (iii) bleaching, to get white fibers by decolorizing their natural creamy appearance, during which oxidant agents, such as sodium hypochlorite, sodium chlorite, and hydrogen peroxide, are always applied; and (iv) mercerizing, realized by immersion in a high concentration of sodium hydroxide solution to improve fabric features, in terms of tensile strength, hygroscopicity, and dye absorbency, brightness, and dimensional stability, occurred through the swelling, the untwisting, and the fiber re-orientation.

In fact, during the mercerizing, the wetted fibers go through a longitudinal shrinkage that can be avoided by elongation and holding the fibers under an applied uniaxial stress. The excess of caustic soda is removed by water washing, (v) dying, and printing to impart the color to the fabric or yarns. All the chromophore agents, such as azo (-N=N-), carbonyl (-C=O), nitro (-N=O), and also amine, carboxyl, sulfonate, and hydroxyl groups are considered water contaminants, because they confer unacceptable color to the wash water [31,32]. Several other chemicals are involved in the dyeing and printing depending on the chemical nature of the fibers: reactive dyes, direct dyes, naphthol dyes, and indigo dyes in the case of cellulose fibers, acid dyes and Lanaset dyes in the case of protein fibers, and finally dispersed dyes, basic dyes, and direct dyes for the synthetic fibers. In order to promote the link between fibers and pigments, binder and polymeric resins should be applied, while the pigments in excess should be removed by washing with detergents, such as alkyl aryl-sulfonates, sulfated alkyl phenol polyglycol, alkylphenol ethoxylates, sodium palmitate, and sodium stearate [31].

Finally, specific finishing treatments should be designed for imparting particular features to the latest products: in the case of water and oil repellency, paraffin (waxes), silicones, fluorocarbon, and stearic-acid melamine may be suggested [33]; for the antibacterial activity metallic salts (Ag+ and Cu2+), triclorosan (2,4,4-hydrophenyl trichloro (II) ether), quaternary ammonium compounds, chitosan, and cyclodextrin are the most common antiseptic products [34]; again, for the flame retardancy halogen based formulations, phosphor, or nitrogen based coating systems, silicone based species are usually implied [35].

In general, all the listed components implied in the fabric production can have different life paths: they can remain attached to the fabric or can evaporate, end up in the wastewater, and be poured into the environment. Although the dilution in large volume of air and water, many of these species may not degrade rapidly, and therefore can be transported at very high distances and accumulate in sediments or organisms (“persistence”); additionally, they can enter the body absorbed through food and skin and accumulate in it (“bio-accumulation”). Many of these species can be considered toxic (“toxicity”) due to the potential or established carcinogenic and/or mutagenic effects, the risk of physical malformation for the fetuses, and the unleashing of allergies of various kinds [4].

A summary scheme of pollutants involved in each operation of the textile industry is reported in the work of Holkar et al. [32]. For example, in the desizing operation, enzymes and waxes are commonly adopted, in scouring fat, soap, surfactants, pectin, and oils are usually applied, for the bleaching hydrogen peroxide, sodium silicate, organic stabilizer, and alkaline pH conditions are necessary; again, metals, salts, alkaline/acid species color, and metals are used; the printing required formaldehyde, urea, solvent, and metals; finally, solvents, waxes, resins, and softeners are needed.

By increasing the complexity of the processes, the machinery technologies, and the automation of the implants, gradually increments of energy consumption correlated to the textile manufacturing have been recorded. In the work of Dhayaneswaran and Ashokkumar [36], it is highlighted that the energy cost is around 15–20% over the production cost, and it stands next to raw material cost, and the power distribution in textile mills including productive and non-productive machines is shown. On the basis of this research, it appears that the highest power is absorbed by ring frame, about 588 KWh, following by speed frame, carding, and compressor.

It can be easily understood that large volumes of water are required for all the described operations that for this reason are referred to the wet process category. In the recent review of Holkar et al. on the textile wastewater treatments, it has been reported that the employment of water for an average sized textiles is about 200 L per kg of fabric processed per day, and finishing treatment generates around 17 to 20 percent of industrial waste water [32].

The implants for the textile production occupy large areas and need huge available spaces. In general, for this reason, the factories are located in low-cost countries, so as to require the transport of goods from the producing countries to the consuming countries. In this way, not only the fuel of non-renewable sources is consumed, but photochemical oxidizing products are also emitted into the air by generating smog. The final products to be transported and sold are protected, preserved, and stored in packaging, generally consisting of non-biodegradable plastic materials, derived from non-renewable sources, paper, aluminum, and cotton, which ends up representing yet another outline of waste and refusal of the textile industry. In the last period, for promoting eco-friendly activities, companies implement the use of recycled or bio-degradable materials for the textile packaging [4,37].

The European Environmental Agency has estimated that clothing, footwear, and household production represented the fifth highest greenhouse production with an amount of released CO_2_ equal to 15–35 ton per ton of produced textiles [38].

Depending on the material nature, the environmental impact of the fiber production is correlated to a complexity of the foresaid multiple factors. Shen et al. [39]. performed a comparative life cycle assessment (LCA) of three types of man-made cellulose fibers (Viscose, Modal, and Tencel), against the conventional textile fibers based on cotton, polyester (PET), and polypropylene (PP). They concluded that the Viscose and Modal could be considered advantageous from an environmental point of view, given the low fossil energy requirements in the pulp and fiber production, while the Tencel resulted in an overall reduction of the energy consumption, chemical use, CO_2_ and SO_2_ emissions, and water consumptions. In the case of cotton, even if it can be considered not an energy-intensive product, the major environmental issues were regarded as the highest fresh water ecotoxicity and terrestrial ecotoxicity, mainly due to pesticide use.

## 4. Textile Recycling: Methods and Developments

**Figure 3:**
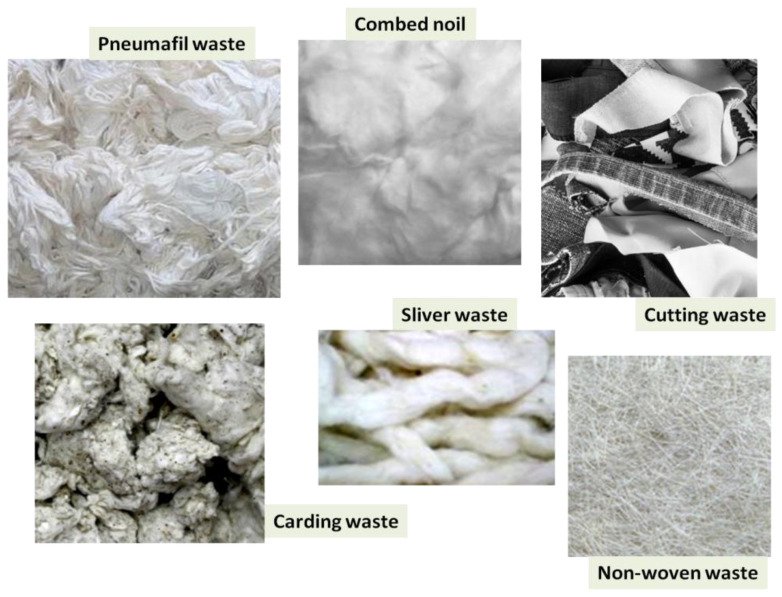
Examples of waste originated by textile world.

An aspect of the textile industry, which in any case has a strong impact on the ecosystem, concerns the large production of solid waste. The types of waste can be classified in function of the relative production: spinning wastes are realized by opening waste, carding waste, sliver waste, roving waste, combed noil, bonda soft waste, and pneumafil waste. Examples of clothing waste are given by knitting waste fiber sulfonate and yarn, woven waste fiber and yarn, woven and knit cutting waste, or also by the use of old clothes; in terms of waste coming from nonwoven production, it can be found thermally and chemically bonded, in lightweight webs, needled webs, and coated and uncoated materials [40]. Finally, a large part of refuses comes from carpet manufacturing. In the work of Wang [41], it has been reported that in the US industries, about 1.4 million tons of fibers (such as nylon (60%), polyolefin (29%), polyester (10%), and wool (0.3%)) have been spent per year for the production of carpets. About every 5–10 years, the old carpets should be replaced by new ones, constituting in this way a quantity of waste equal to 2–3 million tons per year in the U.S. and about 4–6 million tons per year worldwide. The major part of carpet is made of nylon that represents a superior fiber compared to the most common of polyester and polypropylene in terms of features but also in terms of cost. From this perspective, the usefulness, also economic, of recovering a potential resin from the discarded nylon fiber appears to be evident. In the following picture (Figure 3), some examples of textile refuse have been shown.

According to the study on textile waste lifecycle by Domina and Koch [42], all these different forms of solid waste, coming from fabric and apparel, can be grouped into two broad categories: one concerns the unsold merchandise and pre-consumer waste generated by retail that can be easily re-integrated through outlet, jobber, or non-profit organizations; another regards the post-consumer waste of fiber (yarn, fabric scraps, and apparel cuttings) generated by fiber producers, textile mills, fabric, and apparel manufacturers. For these latter, there are three possible disposal routes: (i) to be stored in the landfills, (ii) to be burned in incinerators in order to be converted into energy or powder, or (iii) to be sold to a textile waste recycler and re-converted into reusable goods.

In the landfill, the disposal times are remarkably high, taking approximately weeks or years to decompose natural fibers, and 30–40 years to break synthetic fibers, with a hundred years to their full decomposition. In addition, the delivering of adverse substances into the surrounding soil during the deterioration by possible contamination of groundwater and surface resources and by releasing carbon dioxide and methane, the least considered of the greenhouse gas responsible for global warming, cannot be neglected: this represents a supplementary negative aspect that causes pollution in the surroundings [43].

In general, in terms of reducing the environmental impact compared to incineration and land filling, the operation of reuse and recycling should be preferred; in particular, reuse is considered more beneficial than recycling. In fact, these latter operations could potentially reduce the production of virgin textile fibers and, in the case of reuse, also avoid the further engineering processes required downstream in the textile product life cycle [44].

Generally, most textile garbage is made by both natural and synthetic materials, from one singular system up to the complex ones, generated by the combination of two or more constituents. Clearly, in this latter case, the difficulty in textile recycling grows, and the final quality of the product lowers. In order to correctly operate the fabric recycling, an efficient network should be planned for each type of material that starts from the separation directly during the production phase, according to the differences in pristine components [41].

**Figure 4:**
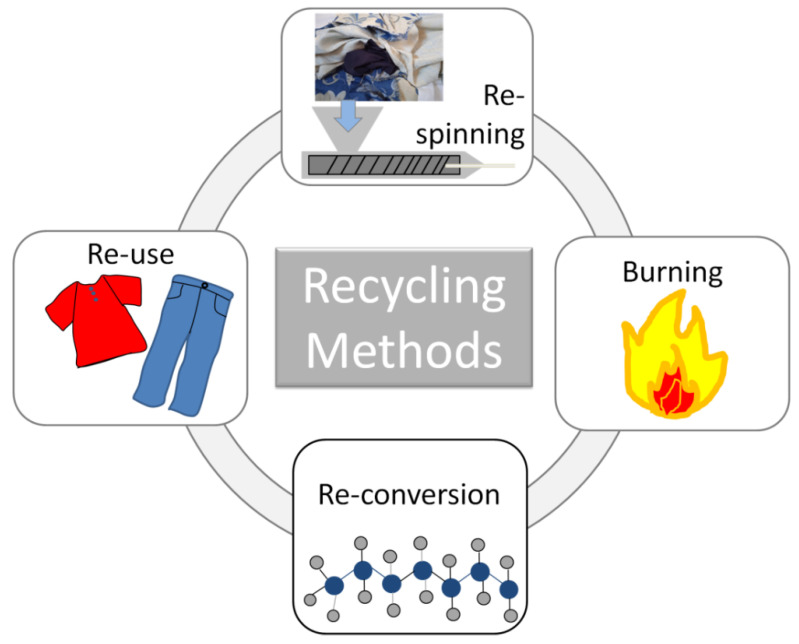
Scheme of the Textile Recycling Technologies.

Recycling technologies are distinguished into four methods, classified as primary, secondary, tertiary, and quaternary approaches [45] (Figure 4): (i) the *primary* one consists in transforming the products in the original form of the industrial recycling scraps; (ii) the *secondary*, i.e., mechanical recycling, involves the cutting and shredding of the textiles for the re-spinning or re-bonding into new yarns and materials, at lower quality and features, both in terms of thermal and mechanical properties, through the melting, re-extruding, and re-blending processes; (iii) *tertiary* technique, also referred as chemical recycling, carries out by processes such as pyrolysis and hydrolysis, for reconverting the products in the pristine constituents (chemicals, monomers, or fuel). This activity consists essentially in a partial or full destruction of the chemical structure of fabric refuse, followed by the reassembly into the original form of the virgin material; (iv) *quaternary* recycling concerns the waste burning for recovering energy and heat.

In the work of Le [46], an overview of both the mechanical and chemical recycling of polyester (PET), nylon, cotton, and wool has been represented in terms of processing steps with recycling flows of post-industrial and post-consumer waste. In a few words, both in the case of polymer-based textiles (i.e., PET and nylon), the mechanical recycling resides in the following steps: the scraps are collected, separated, purified from contaminants or other non-target materials, and reduced into smaller size pieces by crushing, grinding, shredding, or pulling. Then, the so-obtained parts are melted and re-extruded to realize pellets of plastics or filaments and re-worked again into fabric manufacturing processes. In contrast, for the mechanical recycling of natural fibers (i.e., cotton), before the operations of re-spinning and blending, in order to obtain again knitted or weaving or non-woven fabrics, the post-consumer waste should be firstly cleaned, then the external part is separated, and when required, virgin material is added, dust is removed, and the output is carded. As concerns the chemical recycling, in the case of nylon, it can be pursued through three diverse ways, referred as ammonolysis, hydrolysis, and basic/alkaline hydrolysis, to achieve the monomers of caprolactam, or hexamethylenediamine acid (HMDA) and adipic acid, depending on if the starting waste is composed of Nylon 6 or Nylon-6,6. In the case of PET, the chemical recuperation can be carried out through various methods, each of them conducts to the distinct main products: terephthalic acid is obtained by hydrolysis, N, N-Dimethyltryptamine by methanolysis, both polyols and bis(hydroxyethyl)terephthalates are produced by glycolysis, and TPA Amide is formed by ammonolysis. The chemical recovery technology in the case of cotton consists in the dissolution of cellulose through two distinct mechanisms: the former is the depolymerization, made by acid hydrolysis, that leads to glucose monomer, whereas the latter is expressed by Lyocell process or ionic Liquids; while the first terminates with the regenerated man made cellulosic, i.e., synthetic polymers made from natural resources such as Rayon, the second arrives at the formation of recycled cotton products.

Shen et al. (2012) [47] supplied an overview of the LCA, taking into account only non-renewable energy use and greenhouse gas, on the following plastic materials: petrochemical PET, (partially) bio-based PET, recycled PET, and recycled (partially) bio-based PET, by comparing them with PLA (polylactic acid) and Viscose, Modal, and Tencel (i.e., man-made cellulose fibers). Their results demonstrated that both recycled and bio-based polymers were advantageous in terms of environmental pollution, with respect to the single-use petrochemical PET.

Finally, the recent work by Sandin and Peters [44] reviewed the environmental impact of textile recycling and reuse by also exposing scenarios under which these operations are not always totally favorable for the surrounding atmosphere. In fact, if on one hand textile recovery allows us to avoid the production of new products, on the other side, when the replacement rate is low and the manufacturing processes are relatively less polluting, often the customer transport required for the re-collection could overcome the advantages of the prevented manufacture [48].

In Europe (EU), the Environmental Agency has attested an amount of textile waste equal to 5.6 million tons (Mt) until 2013: the respective 20% of the textile waste has been reused or recycled, and 1.5 Mt have been exported outside of the UE, while the 80% was lost [38].

It is interesting to highlight that well-known companies have just incorporated in the plan of production the development of recycled fibers from the textile waste. Leonas [49] reported a series of famous brands implicated in the closed-loop recycling system for fiber and goods produced by recycled fibers. For example, Teijin (Tokyo, Japan) has realized Ecocircle^®^ through a fiber-to-fiber closed loop process based on chemical recycling useful in the men’s and women’s activewear markets. In 2011, Aquafil (Trento, Italy) introduced the Econyl^®^ Regeneration System for recycling Nylon 6 by using both post-consumer waste and pre-consumer waste. Then later, in 2015, in a partnership with Speedo (Nottingham, UK), the same company promoted the recovery of fabric scraps from cut-and-sew manufacturers that have been transformed into a synthetic fiber Econyl^®^. In 2013, H&M (Stockholm, Sweden) launched in its store the “Garment Collecting Initiative” by releasing denim styles made of recycled cotton fiber from the collected clothing. In the 2015, The North Face (San Francisco, CA, USA) released three eco-friendly materials with recycled yarns reprocessed from leftover fabric and recycled plastic bottles for the release of the Denali jackets. Martex Fiber (Spartanburg, SC, USA) gets textile waste and offers recycled cotton textiles (ECO2Cotton^®^) made by post-industrial waste obtained from cut-and-sew operations in apparel manufacturing. Evrnu (Seattle, WA, USA) recovers post-consumer cotton clothing to produce high-standing bio-based fibers. EcoAlf (Barcelona, Spain) converted fishing nets, plastic bottles, tires, and other wastes into jackets, shoes, and bags. Adidas brand [50] also considered the environmental impact of the production line by eliminating the use of hazardous substances, reducing the energy and water consumption, and paying attention to the animal welfare. In addition, this company supports the recycled materials by promoting the recovery of nylon from post-industrial and post-consumer waste, including discarded industrial fishing nets, and also of polyester and polystyrene.

Since 2002, a non-profit organization, called Textile Exchange [51], has been operating to make the valorization of textile waste feasible and sustainable for the enterprises. It promotes strategies to reduce the consumption of raw materials and increase the quality of recycled products. In detail, a regulation called Global Recycled Standard (GRS) has been set up for certifying the companies involving in the use of pre- or post-consumer waste to produce new goods in the textile field. The products must contain more than 20% recycled material to be guaranteed by the GRS certification, including additional criteria for the traceability along the entire production process, for the type of used chemicals, water management, polluting emissions, and energy recovery.

## 5. Recent Applications of Waste Textiles into Organic or Inorganic Matrices

A composite material is constituted by a combination of two or more constituents, mainly referred to as continuous and discontinuous phases. In detail, the first one is called matrix and is represented by polymers, metals, and ceramics, while the second is named reinforcement and, depending on the types, it is distinguished in particulate, fibers (short or filament), and flakes. Usually, the traditional Fiber Reinforced Composites (FRCs) are the most popular, and are made by fibers characterized by a length greater by hundreds of times than the two others sizes [52].

The planning of a composite material allows realizing a system with unique features, in which every phase participates in the final characteristics, proportionally to its volume content (“mixture rule”), so by resulting superior properties to that of each component.

In light of reducing the huge amount of residues generated by the textile industry, scientific research has focused experimental works on the effect of waste textile, as a potential reinforcement, mainly in terms of the mechanical features, for the different types of possible matrices: thermosetting resins, thermoplastics, natural components, and concrete. In the following sections, an overview of recent literature works, performed in the last decade, on the involvement of recycled fibers, embedded both in organic polymer based- matrices and inorganic ones, has been reported.

### 5.1. Thermosetting Resins

Nowadays, the most used fibers in the field of composites are glass fibers (GF), given the respective characteristics of higher strength and stiffness: they are applied for the 87% of the global market of the composites (8.7 million tons). Unfortunately, correlated to the benefits of high-quality reinforcement, GFs involve the main drawbacks of scarce biodegradability and recyclability and of their origin coming from non-renewable resources. Therefore, the need to have replacement of glass fibers, with characteristics related to a higher eco-sustainability, has prompted research into the analysis of different alternatives, such as natural fibers or fibers derived from industrial processing waste. For example, in the work of Umar et al. [53], laminated composites made of cotton fibrous waste have been realized by infiltrating an unsaturated polyester resin and compared in terms of mechanical resistance against the glass fiber-based composites. The used recycled reinforcements have been obtained from two different sources: comber noil waste from spinning and knitting waste. The fiber volume fraction in the composite was maintained at 30%. Final results allowed them to conclude that the studied materials can be applied for low structural applications: the impact strength was comparable to the values obtained for glass fibers composites, but both the tensile and flexural resistance were lower with respect to that of GF-based samples.

Always in the light of minimizing the use of synthetic fibers (GF) by replacing in the composite system, part of their volume, with natural fibers taken from industrial waste, Masood et al. [54] developed hybrid composites, made of an unsaturated polyester resin, and a combination of glass fiber and cotton and jute waste. In this way, the content of the applied synthetic fibers has been restricted, an amount of solid waste has been recovered, and an economic advantage has been derived. In fact, about 16–17% of cotton fibers are thrown away, because, being short, they are not successfully employed in the production of filaments: the developed system was characterized by low strength. Yet, the cotton fibers could be satisfactory in combination with virgin fibers, by gaining advantages on reinforcement effect, and contemporary, by lowering the cost. By analyzing the tensile and flexural properties, the authors concluded that the waste materials could not be an efficient matrix reinforcement compared with glass fibers. However, they could represent, in combination with virgin one, part of the reinforcement of the composite to decrease the cost and to solve the disposal problem, but also obtaining good mechanical features.

In order to enhance the interfacial adhesion between the cotton fibers and epoxy matrix, specific treatment has evaluated been by Baccouch et al. [55]. The cotton was taken from textile waste and shredded into fibers, directly immersed in NaOH solution (alkaline treatment). Then, the so-treated fibers were transformed into non-woven mats by the carding and needle punching process and infused with the epoxy resin. Owing to the chemical modification, an enhancement of filler/matrix interfacial bonding was attested by SEM microscopic observation that led to better mechanical features of composite panels with treated fibers against untreated ones.

The textile waste coming from the tires process has been applied, as a discontinuous phase, in two-component epoxy adhesive. The characterization of the prepared samples revealed that the addition of the recovered fibers from the tire recycling determined an increase of the impact strength, but a worsening of the tensile strength and elongation [56].

Three types of 3D waste denim fiber needled felts, different in areal density, have been incorporated into an epoxy resin by attesting the effect of the characteristics of individual mono-layer fiber web on the mechanical properties of the corresponding composites. From the experimental analysis, it has been verified that the tensile, bending, and compressive resistance was higher in the case of intermediate areal density among the three choices. Yet, given the effective ability of the fibers, regardless of the type, in the load bearing for all cases, the realized composites have been considered satisfactory for the replacement in some particle boards [57].

In the work of Tiuc et al. [58], the textile waste coming from the production of knitted clothing has been introduced in polyurethane foam in order to propose a solution not only to the environmental problem of solid waste accumulation, but also for reducing the noise pollution levels in a unique eco-friendly materials. Concentrations of textile waste, whose constituents were 15% polyamide (nylon), 40% polyacryl, and 45% modal, were introduced in the matrix at up to 50 wt.%. The final results, in terms of sound absorption coefficient, confirmed an improvement of the ability of the developed materials (waste; 40 wt/wt.%) in the reduction of sound equal to twice compared to that of rigid polyurethane foam.

### 5.2. Thermoplastic Polymers

Serra et al. [59] proposed the use of waste dyed cotton flocks, in a content of 30 and 40 wt.%, as reinforcement for polypropylene-based composites. In order to increase the tensile strength of the tested samples, formulations realized by adding maleic anhydride (MAPP), in a content ranging between 0–8 wt.%, have also been tested. Contrary to the expectation, in the case of uncoupled samples, a higher tensile strength has been verified. This result has been attributed to the effect on the dye on the fiber/matrix interphase. Indeed, the presence of tincture in the considered byproduct should be inevitably taken into account, since in the textile process, the used yarns are always subjected to dyeing. In order to better understand the effect of coloring agent on the features of the final materials, a comparison with the virgin fibers has been also investigated. By analyzing the data, it has been concluded that the dying agents could increase the hydrophobicity of cotton fibers, and consequently the respective affinity with the polypropylene resin, by leading to a greater mechanical resistance for composites, essentially without compatibilizer.

In the case of the study by Araújo et al. [60], the cotton fibers, obtained from a low-value textile waste, were cut with common scissors and treated with 2.5% sodium hypochlorite for removing the dye removal and bleaching. Then, the so-prepared fibers were chemically modified on the surface using acetylation or silanization method in order to improve the interaction between the fibers and the polypropylene matrix. The FTIR results confirmed the chemical modification of fibers by acetylation and silanization. The mechanical properties, analyzed through tensile tests, allowed them to verify the effectiveness of treated fibers reinforced in the features of the pristine resin; in fact, the composites showed higher storage modulus, Young’s modulus, and tensile strength, when compared to neat PP. The authors reported an increment of the tensile modulus from about 1300 MPa, to approximately 1700 MPa in the case of adding the 5 wt/wt.% of fibers modified by silanization methods, and equal to 2100 MPa for the incorporation of 20 wt/wt.% of recycled fibers modified by acetylation. Yet, at an equal fiber amount, interesting similar results seemed to be achieved also in the case of recycled untreated fibers, compared to the treated ones. By thermogravimetric measurements, it seemed that the presence of fibers, both treated and untreated, led to an improvement of intermediate stability with respect to matrix and fiber, while calorimetric features attested to a reduction of crystallinity degree due to the fillers addition. Compared to the untreated fibers, stronger interfacial adhesion was established by scanning electron microscopy (SEM) for treated samples. The authors suggested the developed composites for the automotive sector.

Always by involving the polypropylene, as matrix, but in the form of fibrillated textile waste, Echeverria et al. [61] investigated assorted end-of-life textiles, as a low-carbon alternative feedstock, for realizing FRCs useful in the building applications. They promoted a cost-effective multi-stage cascading process in which additives, pretreatment, and coating were avoided for reducing the applied chemicals and the environmental impact: the post-consumer textile waste was recovered through recycling, reformed into reusable material, and transformed at the end of their life when it reached the maximum limit for the recycling. Homogeneous microfibrils of polymers based on thermoplastics (i.e., polyester, acrylic), ligno-cellulosic (i.e., cotton), and proteins (i.e., wool) constituted the main filler phase, while, in addition, residual fine wood fibers have been introduced as secondary filler. For optimizing the interfacial adhesion between matrix and filler, the maleic anhydride grafted polypropylene coupling agent has been used in percentage equal to 6 wt.%. Composites were prepared by isothermal hot-compression molding, by investigating not only the mechanical characteristics, but also moisture absorption, fire resistance, and surface roughness. The highest performance, intended as a flexural strength of 3.9 MPa and water absorption of 2.4%, has been obtained by introducing an amount of polypropylene equal to 40 wt.%, while for optimizing the fire resistance, the presence of the secondary filler has been demonstrated to be essential, maybe due to the involvement of sodium and calcium content. The authors concluded that, depending on the flexural characteristics, the developed prototypes may be usefully optimized for flooring, walling, and division systems (loading-bear systems) or interior linings, such as ceilings or acoustic absorbers (non-loading bear systems).

### 5.3. Natural Matrices

Recycled textile fibers derived from recycled second-hand jeans were used in combination with ignifuged miscanthus fibers for developing new insulating biocomposites constituted by chitosan as polysaccharide-based binder and aluminum trihydroxide (ATH) as filler. Firstly, binary systems of chitosan and (ATH) have been tested by showing significant improvement in the thermal behavior by increasing the filler content. Since for these compounds, the mechanical performance has undergone to a reduction, the realization of hybrid material by introducing miscanthus/recycled fibers have been investigated. These samples have been prepared through thermocompression process. The final results, both in terms of mechanical, thermal, and fire behavior, allowed us to conclude this is a promising application of these systems for bio-based non-flammable and insulating building materials [62].

Wood fibers and textile waste fibers have been used in preparing biocomposites through a natural polysaccharide, derived from a brown algae able, to form a stable gel that acted as an adhesive binder: the sodium alginate. A homogeneous mixture of wood/waste fibers, at different compositions (100/0, 50/50, 60/40, 70/30, and 0/100 in weight) was soaked progressively in a solution of sodium alginate, also by adding a crosslinking agent. Samples were obtained by thermocompression and tested in terms of thermal (diffusivity, effusivity, and conductivity) and mechanical properties (compressive and bending strength). The introduction of textile fibers in the developed hybrid bio-materials contributed to increasing the plasticity of the final products that could be balanced trough a higher rigidity, achieved by the addition of the crosslinker. The best results were shown in the case of 60/40 *w*/*w* wood/textile waste with a glutaraldehyde crosslinking agent by displaying the maximal mechanical strength of 0.84 MPa under bending and 0.44 MPa under compression. All the biocomposites possessed insulator features, with a thermal conductivity in the range of 0.078–0.089 W/m/K. Therefore, it can be concluded that the designed hybrid material can be located between insulation and rigid panels for building [63].

In the work of Rubino et al. [64], composite panels have been made from wool waste fibers in the form of cut fabrics, bound through two different polysaccharides: a chitosan solution from crustacean by-products, and a gum Arabic solution from the Acacia tree. The material has been designed for potential building construction by experimenting with the sound absorbing and thermal insulating properties. The prepared samples revealed a thermal conductivity very similar to the traditional building materials, with values in the range of 0.049 and 0.060 W/(m K), depending on own density and porosity. In particular, these two latter aspects also affected the air flow resistivity by leading to better sound absorption in the case of more porous structures. Ignitability testing demonstrated the best behavior with very limited flame propagation, lesser smoke, and droplets of melted material, in the case of pressed wool. Both binders (Arabic gum and chitosan) have been proved to be equivalent in terms of final performance.

In order to realize wearable strain sensors, waste cotton fabrics were heated until carbonized, and then impregnated with natural rubber latex (NRL) using three methods, i.e., vacuum bagging, negative pressure adsorption, and drop coating. After carbonization, the knitted texture of the cotton was maintained, but the mechanical characteristics of the pristine fibers were lowered. The fiber treatment with the latex binder contributed to an increase of the tensile strength by increasing the NRL content in the composites. The developed materials have been also tested as a wearable flexible device for monitoring the finger bending and the muscle contraction. In this case, the relative resistance measured during tensile test increased as the strain increased by showing stability under repeated stretch–release cycles [65].

### 5.4. Hybrids

The study of Chuang et al. [66] aimed to recycle high strength polyester (PET) waste fibers, in view of possible recovery for cheaper high-performance materials useful in the fields of protective cloths or geotextiles. Recycled high-strength PET selvages were combined with low melting and woven fabrics in hybrid fibrous planks by a thermal process. In the preparation of the specimens, carbon, aramid, and basalt woven fabrics have been used as reinforcement, while the low melting point PET (LMPET) staple fibers have been applied as adhesive between the two phases. The high-strength selvages were mixed with the low melting point fibers and processed in the needle punching machine. Air permeability, tensile, tear, bursting, and stab resistance tests have been performed on the developed materials. The results displayed that the recycled PET fabrics remained characterized by a higher strength. Benefits on the mechanical resistance arise from the combination of woven and non-woven systems by providing good stab behavior and a strengthening of the puncturing features in hybrid-fabric fibrous planks.

In the light of two environmental pollution problems, urban noise and waste fibers, Echeverria et al. [67] proposed hybrid Fiber Reinforced Composite (HFRC), useful in the sound absorption building applications, realized by natural and synthetic fibrous polymeric materials, coming from the post-consumer and end-of-life waste streams. Different formulations have been designed for constituting three different hybrid isotropic series: textile fleece, mattresses foams, and wood-plastics. The used sustainable materials, applied in the manufacturing of the prototype panels, were: (i) assorted polymer fibers, whose main constituents were cellulose, protein, and plastic, derived from second hand clothes blends; (ii) polypropylene textile, used as matrix in all three series of composites, was taken from packaging waste, as woven sacks and shopping bags, (iii) polypropylene particulate powder, utilized as a matrix in the wood plastic type obtained from waste food containers; (iv) organic marine fibers, mainly derived from Kelp brown algae blades and bivalve mollusc shells, were hand-collected. The experimental results showed that all three series of developed HFRC materials presented low sound absorption performance at mid frequencies and a higher absorption coefficient at higher frequency ranges. The peak sound absorption of 78% and 75% was verified in correspondence of wool–acrylic and wool–polyester blends, respectively.

An innovative eco-friendly material, interesting for the applications in buildings and constructions, derived by agricultural and industrial waste has been proposed in the study of Muthuraj et al. [68]. Rice husk, wheat husk, wood fibers, and textile waste fibers were used, in combination, as fibers in biopolymer matrices of poly (butylene adipate-co-terephthalate)/poly (lactic acid). Composites, containing 13.5 ± 2% in binder content, were realized by the hot pressing method. The morphological characterization led to the verification of a better compatibility between the binder and fibers of wood and textile fibers rather than with wheat and rice husks. The prepared systems showed good thermal stability up to 250 °C. Among the developed biocomposites, the wood fiber based systems possessed the highest compressive modulus and strength, while the rice husks based ones displayed the better insulation features and lower water adsorption.

### 5.5. Concrete Materials

Concrete materials possessed the advantages of easy manufacturing process, high strength, and durability. Unfortunately, their drawback is represented by a poor resistance against aggressive conditions, such as seawaters and soils that contain sulfates due to industrial sewage and drainage wastewater. In this perspective, in the work of Mohammadhosseini et al. [69], the inclusion of polypropylene, recycled by waste carpets, and palm oil fuel ash components has been investigated for preventing crack formation and deterioration. In this way, not only benefits in the final products have been expected, but also a help in decreasing landfill area and in the preserving the environment pollution has been promoted. From the experimental results, the authors concluded that the addition of the waste fibers, depending on the content, decreased the workability of the fresh concrete, diminished the compressive strength of the final manufacture, but increased the respective tensile strength, and resulted in a reduction of crack formation for the bridging effect. As concerning the aggressive environment, the performance of the concrete containing carpet fibers and palm oil fuel ash have revealed a good resistance to the acid attack. In the work of Chen et al. [70], monofilament polypropylene fibers and fiber bundles have been obtained by waste polymer textile bags and used as reinforcement in the cemented clay admixture. The laboratory experiments confirmed that these additives, depending on the fiber type, the corresponding content, and length, could effectively increase the strength and ductility of the final products.

Besides polypropylene, recycled polyamide fibers have also been recovered by waste textiles and introduced in cementitious matrices. For example the work of Spadea et al. [71] analyzed the compression and bending features of cementitious mortars reinforced with recycled nylon fibers, taken from waste fishing nets. In the past, the finishing nets were made by biodegradable materials such as cotton and linen; today, the most common constituents are made of plastics. In the view of the protection of the sea environment, and also for reducing the cost of the products, the recycling operation for this synthetic production is considered extremely important. The experimental results showed an increase of the tensile strength (up to +35%) and toughness (up to 13 times greater) of the nylon reinforced mortar respect with the unreinforced material. This outcome was considered by the authors to be a sign of the environmental and mechanical potential of recycled nylon fibers for sustainable cement. Not only synthetic fibers, but also natural ones made of recycled cotton or jute have been studied in terms of reinforcing effect for the construction materials. Peña-Pichard et al. [72] developed polyester concrete with waste cotton fibers from blue jeans. Lower compressive strength but higher flexural properties have been verified for concretes with 1.0 wt.% of waste cotton fibers, demonstrating a superior elasticity acquired in the final samples. Jute fibers obtained by waste bags have been introduced in different proportions up to 20 wt.%in the Portland cement by Ferrandez-García [73] for realizing prefabricated panels for interior partitions. Starch has been also introduced in the formulations as plasticizer. Characterizations of the density, swelling thickness, internal bonding, flexural strength, and thermal conductivity have been performed on the prepared specimens. The authors concluded that jute–cement composite panels can be manufactured satisfying the European standard for the use in the construction of buildings as partitions, interior divisions, and thermal insulators.

In the following table (Table 2), recent papers on the recycled fibers by textile waste applied to produce composite materials based on thermosetting, thermoplastic, natural, and concrete matrices have been summarized.

## 6. Discussion

This work represented an overview of the environmental issues related to the textile industry, by proposing a positive perspective in the reuse and recovering of the recycled fibers in the field of the composites materials for different applications.

The recycling technologies consist in mechanical and chemical methods by which they can take benefits from the textile waste, and it can reduce the volumes of the solid waste ending up in the landfills. During these operations, the garbage is transformed into industrial scraps melted and spun again into two filaments, reconverted in pristine constituents, or burned for recovering heat and energy. The recycling operations arise from the need to limit the environmental impact of textile production. In fact, harmful chemicals, water and energy consumption, air emission, and the involvement of the transportation means and packaging have been all considered polluting aspects during the typical progressive phase of the manufacturing process. Pesticides, insecticides, and fertilizers are used in the raw material treatments for the cellulose- and protein-based natural fibers, whereas monomers and catalysts are applied for the preparation of the synthetic polymer-based filaments. Then, following the fabric production, sizing chemicals to improve the absorbent capacity; oxidizing agents to improve strength, hygroscopicity, dye absorbency and brightness; pigment, chromophore agents, and dyes to improve the color, and finally specific finishing treatment for imparting the water and oil repellency and antibacterial features should be taken into account, along with the question related to the water and energy consumption in each developing phase.

Yet, some studies on the LCA cycles highlighted that if, on the one hand, the textile recovery allows a less polluting textile manufacture, on the other hand, it requires an elevated customer transport for the re-collection, certainly detrimental for the surrounding atmosphere. However, it can be positively underlined that many well-known brands have already adopted company policy strategies aimed towards an eco-friendly production process, starting from recycled fibers.

In this scenario, alternative approaches have been investigated in the recent literature by incorporating the recycled fibers from the textile waste into organic and inorganic matrices for enhancing the characteristics of the final products and, at the same time, limiting the environmental concerns of the textile fabrication.

For example, in the case of thermosetting resins, mainly epoxy, but also polyurethane foam and polyester based synthetic fibers of polyamide, and natural fibers of cotton taken from defective or knitted cloths, have been added for reducing the noise in structural applications or reinforcing automotive components. In detail, cotton fibrous waste, also in combination with the jute fibers, has been infiltrated by an unsaturated polyester resin for realizing laminated composites, endowed with mechanical features, almost comparable with glass fiber-based composites. In this way, besides a reinforcing effect, a cost reduction was also derived. Then, recycled cotton fibers, also treated in alkaline solution, were used in an epoxy resin by determining a remarkable enhancement of the mechanical resistance in the final materials in light of the load bearing applications. Analogously, for the same materials, the water absorption test confirmed a lower diffusion coefficient values as compared with pine, oak, and linden wood by making them suitable for timber replacement in the furniture items.

In the case of thermoplastics, common polypropylene, or also recycled polyethylene and polyamide, or even biopolymers, were mixed primarily with cotton fibers taken from residue flocks, not long enough to be spun again, or from denim not approved for the poor quality. Polypropylene has been tested with waste dyed cotton flocks, by verifying the effect of coloring agent, and of a compatibilizing agent, on the mechanical and interfacial features of the respective products. Final results allowed to attest that dyeing agent improved the compatibility between the fiber and polymer by resulting in a higher mechanical resistance compared to the systems containing the compatibilizer. Always by using the same polyolefin, as a matrix, the effect of recycled silk and wool has been attested on the thermal conductivity, mechanical performance, and water absorption of the ultimate compounds, from the perspective of realizing materials useful in the printed circuit boards. It was found that the higher the fibers content in the polypropylene, the lower the thermal conductivity and higher the water absorption in the corresponding compounds.

Formulations based on natural constituents have been realized with textile recycled fibers of cotton or wool for the purpose of increasing the thermal insulation and noise control in the construction industry. In particular, sodium alginate has been adopted as an adhesive for bonding wood fibers and textile waste though thermo-compression. The so-prepared specimens have been tested in terms of thermal and mechanical characteristics by showing good performance for insulator or rigid panels. Another two binders, made by Chitosan and Arabic gum, were experimented with together with the wool waste fibers for obtaining materials useful in the construction industry. The sound absorption, the thermal conductivity, and ignitability behavior of the corresponding systems have been attested in function of the structure porosity. More porous structure possessed lower thermal conductivity but higher resistance to the air flow ability and for this reason led to a good sound absorption feature. Then, natural rubber latex was used for impregnating cotton fabric waste, treated by heat until carbonization. The corresponding behavior has been attested in terms of the tensile strength and the electrical resistance, measured by increasing the strain, during the mechanical test, to reproduce the finger bending and the muscle contraction in the case of a wearable flexible device.

Hybrid compositions made of polymer matrix (both thermosetting and thermoplastic) have been prepared by mixing two or more fillers, one of them originated by the textile waste for a wide variety of applications. For example, recycled high strength polyester was combined with woven fabrics based on carbon, aramid, and basalt constituents, in hybrid fibrous planks, potentially applicable in protective cloths or the geotextiles field, by testing the permeability behavior and the mechanical performance. Other formulations, involving polypropylene textile waste, assorted polymer, fibers, and polypropylene particulate powder from waste food containers have been studied by producing prototype panels useful in the sound absorption for building applications. In the light of the same intent, rice husk, wheat husk, wood fibers derived from agricultural waste, and textile waste fibers, have been combined in a unique innovative eco-friendly material, and tested in terms of insulation features, water adsorption, and compressive strength. Depending on the concentration of each fiber, good compressive strength, or better insulation features and lower water adsorption could be derived in the final systems.

Finally, in the concrete materials, as cement–clay admixtures, cementitious mortars, or Portland cement, fibers of polypropylene, derived from textile bags, or of nylon, taken by finishing nets, have been added for the improvement of the soil paving or for the protection of sea environment, respectively. In detail, recycled polypropylene by waste carpets has been tested with palm oil fuel ash for preventing the crack formation and deterioration in concrete. Even if the polymer addition decreases the workability of the fresh constituents and the mechanical performance of the final products, good resistance to the acid attack and decrement in crack triggering have been shown. Then, fibers of polyamide have also been recovered from waste finishing nets and inserted efficiently in cementitious mortars, by obtaining increment of tensile strength and toughness in the ultimate product compared to the basic ones. Additionally, natural fibers of cotton or jute have been investigated within the cementitious matrices. Their application has been considered useful for improving the elasticity of the final samples in the interior building partitions by meeting the European standard.

## 7. Conclusions

In this work, a useful perspective for the recycled fibers derived from the textile waste has been presented by illustrating the corresponding applications in the recent scientific studies. Nowadays, many are the efforts aimed to limit the environmental damage of the textile production: well-known brands have just supported strategies devoted to the use of recycled materials, reduction of the solid waste, and wastage of the raw constituents; non-profit organizations were born for certifying companies that use pre- or post-consumer waste for their products; and finally, the scientific research has investigated the potential valorization of the textile waste in multifunctional materials by reducing the ecological footprint with the circular economy. The produced fibers from textile waste have been efficiently incorporated in organic and inorganic mixtures, with both natural and/or synthetic components, by testing mainly mechanical, acoustic, thermal, and electrical features. Different parameters have been recognized to affect the final properties of the developed products: the presence of coloring or compatibilizing agent, fiber treatment by heat or chemicals, structure compactness and corresponding porosity, fiber type, and content. In particular, recycled fibers, mostly based on cotton, have been added in thermosetting resins as reinforcement for structural applications, also with the intent to replace the traditional harmful glass fibers. In the thermoplastics, recycled cotton was introduced for obtaining a reinforcing effect in view of automotive components, while the silk and wool were applied for their insulating features in view of printed circuit boards. A particular employment in the field of protective cloths has been proposed: in the case of recycled Kevlar or High Strength polyester, the cutting and mechanical resistance of both materials being well-known. Natural constituents, made of chitosan, sodium alginate, as well as natural rubber, have been used as a binder for natural recycled fibers of cotton or wool for realizing buildings, walling, interior linings, and division systems, by exploiting the fibers’ characteristics in terms of sound absorption, noise control, and thermal insulation. Then, waste bags, finishing nets, and waste carpets made of polypropylene or nylon have been recovered and used in cementitious mortars for improving the corrosion resistance and avoiding crack formation or for augmenting the tensile strength and the toughness.

## Figures and Tables

**Table 1:** Examples of recent research studies based on the incorporation of fibers in the polymer matrices for a large range of applications.

Matrices	Fibers	Applications	Ref.
Thermosettings-based Reinforced Composites			
Epoxy Resin	Jute fibers/wood dust	Lightweight and medium load applications	Dinesh, et al., 2019 [6]
Epoxy Resin	Glass Fibers	Military applications and ballistic protection	Patterson, et al., 2020 [7]
Epoxy Resin	Flax Fibers	Eco-friendly materials with tensile and wear characteristics	Kumar, et al., 2020 [8]
Thermoplastics-based Reinforced Composites			
Poly(lactic acid)	Sisal fibers	High-performance biodegradable composites	Huang, et al., 2015 [9]
Poly(lactic acid)	Aramid fibers	Fused Deposition Model	Bettini, et al., 2017 [10]
Polypropylene	Hemp fibers	Semi-structural automotive components, such as seat back, door inserts, underbody panels, and instrument panels	Sullins, et al.2017 [11]
Poly(lactic acid)/Thermoplastic Starch	Cotton fibers	Structural reinforced components and green packaging	De Macedo, et al., 2019 [12]
Polypropylene Polyamide 6 Polyphenylene sulfide	Carbon fiber	Aircraft structural applications	Taketa, et al., 2020 [13]
Nylon	Carbon Fibers	3D printings	Liu, et al., 2020 [14]
Poly(lactic acid)	Cellulose fibers	Polymer recyling for additive manifacturing	Patti, et al., 2020 [15]
Hybrids			
Epoxy Resin	Hemp fibers Glass fibers	Rotorcraft interiors	Scarponi, et al., 2015 [16]
Thermoplastic polyurethane	Sugar palm fibers/Glass fibers	Building, automotive part, construction for environmental awareness	Atiqah, et al., 2018 [17]
Epoxy Resin	Basalt Fibers/Organoclay	Automotive and Aircraft	Bulut, et al., 2020 [18]
Concrete- based Composites			
Asphalt Concrete	Steel fiber and graphite	To produce electrically conductive asphalt concrete	Wang, et al., 2016 [19]
Concrete	Sisal fibers as internal reinforcement Jute fibers as external reinforcement	Sustainable construction	Tan, et al., 2017 [20]
Plain cement concrete	Glass Fibers	To improve the overall mechanical perfomance	Babar, et al., 2019 [21]
Asphalt concrete	Kenaf and Carbon fibers	To enhance the fracture toughness	Pirmohammad, et al., 2020 [22]
Light weight concrete	Jute, Coconut, Sugar cane, Sisal, Basalt	Thermally efficient building materials	Asim et al., 2020 [23]

**Table 2:** Examples of recent research studies based on the incorporation of fibers in the polymer matrices for a large range of applications.

Matrices		Fillers	Content	Textile Source	Applications	Year
Thermosettings -based Reinforced Composites						
Polyurethane foam	Polyamide, polyacril and modal		50 wt.%	Textile waste from the production of knitted clothing	Materials to reduce noise pollution levels	2016 [58]
Two component epoxy adhesive-	Polyamide microfibers		5 wt.%	Waste from the process of the tyres recycling	Waste disposal problems	2016 [56]
Unsaturated polyester resin	Cotton		30 vol.%	Combed noil waste from spinning and the knitting waste	Low strength structural applications	2017 [53]
Unsaturated polyester resin	Cotton, jute and glass Fibers		30 vol.%	Textile Industry waste	Minimizing the use of synthetic Glass fibers	2018 [54]
Epoxy resin	3D denim-cotton fiber needled felts		~30 wt.%	Waste jeans	Construction fields, such as particle boards, plywood and flooring	2020 [57]
Epoxy resin	Cotton fibers treated in alkaline solution and trasformed into nonwoven mat		1.5 M	100% cotton waste supplied by SITEX(Tunisia)	Lightness and good mechanical performances automotive andbuilding industries	2020 [55]
Epoxy resin	Cotton		40 vol.%	Cutting waste in garment making and defective fabrics	Replacement of timber for furniture materials and automotive components	2020 [74]
Thermoplastics-based Reinforced Composites						
Recycled Polyamide (Nylon 6,6)		Micro-Cellulose	30 wt.%	Carpet waste to recycle the nylon matrix	Automotive interior and exterior parts	2012 [75]
Polypropylene		Silk/Wool	50 wt.%	Silk selvedge waste	Electrical insulating material in printed circuit boards	2013 [76]
Recycled Polyethylene		Cotton	30 wt.%	Textile waste cotton fabrics	Green composites	2017 [77]
Polypropylene		Cotton	40 wt.%	Flocks residues, treated with a reactive dye, from textile industry and with not enough length for spinning	Green composites	2017 [59]
Polypropylene		Cotton	20 wt.%	Denim cotton yarns, not approved by quality standard and sold as low-value waste	Automotive	2017 [60]
Poly(lactic acid)		Hydrolyzed cotton fibers surface modified with various silane	5 wt.%	Denim cotton waste	green composites	2018 [78]
Bio-based polyethylene terephthalate		Cotton	10 wt.%	Linter of recycled cotton	Environmentally friendly and cost-effective composite materials	2019 [79]
Polyacrylonitrile		Alpaca fibers	30 wt.%	Waste alpaca fibers	Reduction of the ecological footprint and supporting the circular economy	2019 [80]
Natural matrices-based Reinforced composites						
Sodium alginate		Wood and textile waste fibers	/	Recycled jeans	Insulation and rigid panels for building.	2018 [63]
Chitosan		Miscanthus fibers/recycled textile fiber/Aluminum trihydroxide	92 wt.%	Recycled second-hand jeans	Non-flammable and insulating building materials	2019 [62]
Vegetable (Gum Arabic) and animal (Chitosan) resources		Wool	40 wt.%	Discarded shreds resulting from the manufacturing process of an Italian clothing company	Materials used in the construction industry for thermal insulation and noise control	2019 [64]
Natural rubber latex		Cotton	22.4 wt.%	Knitted fabrics from cardigans	Flexible strain sensor	2019 [65]
Natural rubber		94% Cotton/6% polyester	80–90 wt.%	100% knitted cotton fabric strips and 100% polyester threads sourced from the overlock machines	Sound insulation materials	2021 [81]
Hybrids						
PP resin and styrene accelerator.		Loose fibrous material	40 wt.%	Threads, rags, and woven cloth scraps	Green composites	2014 [82]
Epoxy resin		Polyamide fibers and rubber particles	12 wt.%	Process of tyres recyclation	To set a possible utilization of unsorted textile waste from the process of the tyres recycling	2015 [83]
Low-density polyethylene		Glass/Cotton fibers	35 wt.%	Chopped waste fabrics	outdoor products Outdoor products	2017 [84]
Polypropylene		Sisal/Recycled E glass Sisal/Recycled Carbon fibres	42 wt.%	Waste parts of final fabrics textile manifacturing	Semi-structural applications	2018 [85]
Fibrillated polypropylene textile wastee		Cotton, wool, acrylic, polyester, polypropylene, nylon and elastane	/	Local municipal solid waste	Flooring, walling and division systems (loading-bear systems) or interior linings, such as ceilings acoustic absorbers (non-loading bear systems)	2019 [61]
Recycled Polypropylene Polypropylene particulate powder Latex and polyurethane mattresses foams		Cellulose, protein and plastic Mixed wood sawdust Organic marinefibres	/	Assorted polymer fibers by second hand clothes blends, Polypropylene from woven sacks and shopping bags, Latex and polyurethane mattresses foams by a recycling company	Sound absorption building applications	2019 [67]
PLA/PBAT blend (55/45 wt.%)		Rice husk, wheat husk, wood fibers and textile fibers	86.5 wt.%	Garments recycling company, with a minimum of 70% cotton content	Buildings and constructions	2019 [68]
Polyester staple fibers		Fibers/Woven Aramid	90 wt.%	Recycled Kevlar woven selvage	Protective cloths	2019 [86]
Recycled polyester Low melting point polyester		Carbon, basalt and aramid plain woven fabrics	/	High Strength polyester selvages	Protective clothing field and geotextiles field	2019 [66]
Epoxy resin		Cotton fibers graphite oxide nanoparticles	40 vol.%	Textile waste called as shoddy	Furniture materials and some visible and non-visible automotive components	2020 [87]
Recycled Polypropylene in form of particles and fibers		Lignocellulosic filler-phase	40 wt.%	Particulate PP from discarded assorted food packaging, and the fibrous textile PP	Multifunctional sustainable building materials from heterogeneous complex waste mixtures	2020 [88]
Concrete Materials						
Cement–clay admixture		Polypropylene fibers	2.5 wt.%	Waste bags	Compacted-pavement base/subbases	2015 [70]
Cementitious mortars		Nylon fibers	1.5 wt.%	Fishing nets	To protect the sea environment	2015 [71]
Foamed Concrete		Textile fiber wastes	5 wt.%	Industrial textile waste	To increase the energy efficiency of buildings	2017 [89]
Portland cement		Polypropylene fibers	1.25 vol.%	Waste carpet fibers	New alternative concrete composite exposed to corrosive ambient	2018 [69]
Polyester concrete		Cotton fibers	1.4 wt.%	Blue-jeans waste	Solution to the environmental problem of textile waste	2018 [72]
Portland cement/Starch		Jute fibers	20 wt.%	Waste jute bags	Prefabricated panels for interior partitions	2020 [73]

## Data Availability

No new data were created or analyzed in this study.
